# Evaluation of Pharmacology and Pathophysiology Knowledge of Epilepsy among Senior Pharmacy Students: A Single Center Experience

**DOI:** 10.3390/medicina59050848

**Published:** 2023-04-28

**Authors:** Nasser M. Alorfi, Ahmed M. Ashour, Hanouf S. Bafhaid, Fahad S. Alshehri

**Affiliations:** Department of Pharmacology and Toxicology, College of Pharmacy, Umm Al-Qura University, Makkah 24382, Saudi Arabia

**Keywords:** epilepsy, pharmacology, pathophysiology, neuroscience, antiseizures

## Abstract

*Background and Objectives:* Epilepsy is a chronic disease that causes substantial morbidity and mortality. Pharmacists represent an integral role in managing patients with epilepsy. The aim of this study was to evaluate the level of knowledge about the pharmacology and pathophysiology of epilepsy among senior pharmacy students. *Materials and Methods:* Cross-sectional study using a designed questionnaire to measure the pharmacological and physiological knowledge of senior pharmacy students regarding epilepsy who are studying at Umm Al-Qura University, Makkah, Saudi Arabia, from August to October 2022. *Results:* A total of 211 senior clinical pharmacy students responded to the questionnaire. The majority of the respondents were 4th year pharmacy students. The numbers of female and male participants were equal (106 and 105 students, respectively). The participants represented an acceptable level of knowledge about the pathophysiology aspects of epilepsy, with a mean total score of 6.22 ± 1.9 out of a maximum score of 10. The respondents reported that epilepsy could be due to genetic predisposition combined with environmental conditions (80.1%) or brain stroke (17.1%). Regarding the respondent knowledge about the pharmacology of epilepsy, the total score was 4.6 ± 2.1 (maximum attainable score: 9). *Conclusions:* The majority of pharmacy students had knowledge about the pathophysiology concept of the disease; however, low knowledge was shown by the respondents regarding the pharmacology of epilepsy. Thus, there is a need to identify better strategies to improve students’ education.

## 1. Introduction

Epilepsy is a chronic disease associated with stigma, seizure-related disability, high mortality rates, psychiatric comorbidity, and high economic costs [1,2]. Epilepsy is a common neurological disease in Saudi Arabia; it is reported that 0.654% of Saudis have this disease [3]. The International League Against Epilepsy (ILAE) has published several guidelines related to the diagnosis, treatment, and management of epilepsy. Almost 5 million people worldwide are diagnosed with epilepsy annually, according to the world health organization (2022). The prevalence of active epilepsy (defined as having at least one unprovoked seizure in the past five years or currently taking antiseizure medication) was 7.6 per 1000 people globally (ILAE, 2020). The onset of epilepsy is most common during childhood, with about 75% of cases developing during this period. This is likely due to the fact that the developing brain is more vulnerable to seizures [4]. The major causes associated with the development of epilepsy in all ages are congenital developmental and genetic conditions [5].

In people with epilepsy, the balance between excitation and inhibition of neurons in the brain is disrupted, leading to abnormal patterns of electrical activity [6,7]. This can cause neurons to fire in a synchronized and excessive manner, creating a wave of electrical activity that can spread through the brain and cause a seizure [8]. The specific pathology of epilepsy can vary depending on the type and cause of the condition. The disease involves repeated, intermittent, and unpredictable seizures of the cerebral cortex. While subcortical areas are less studied and understood than the cortex in epilepsy, recent research has shown that they may play a more significant role in some types of epilepsy than previously thought [9]. For example, some types of focal epilepsy, which originate in a specific area of the brain, may involve subcortical structures in addition to the cortex [10]. According to the focus and spread of discharges, unprovoked recurrent epileptic seizures may be classified as generalized, focal, and epileptic spasms [11]. Focal seizures arise from a localized area of the brain, while generalized seizures usually begin focally and then spread to include large areas of the brain [12,13].

The main aim of epilepsy treatment is to achieve complete remission of seizures [14]. Antiseizure drugs act generically to inhibit the rapid firing and repetition of neuronal action potential that characterizes seizures. They are mainly designed to reduce excitatory neurotransmitters, such as glutamate, and stimulate inhibitory neurotransmitters, such as gamma amino butyric acid (GABA) [5,15]. Currently, there are approximately 30 pharmacological treatments for seizures that have been approved by the United States Food and Drug Administration (FDA) [16]. Most antiseizure drugs suppress seizures in up to two-thirds of all patients, but they do not affect long-term outcomes [17].

The first (old) generation of antiseizure drugs exhibits a strong efficacy profile but is associated with unfavorable pharmacokinetic and multiple-drug interactions [18]. The second (new) generation of antiseizure medications demonstrated less potency compared to the older generation [14]. However, the newer generation elicits synergistic effects when used in combination with other antiseizure drugs as well as induces fewer adverse effects [14]. An attempt to develop novel disease-modifying agents is currently under preclinical and clinical development [5].

The non-pharmacological approach is also proposed for epilepsy control, including ketogenic diets. It was postulated that a keto diet with high fat and low carbohydrate content improves seizure control by modulating neurotransmitter release, such as GABA, the major inhibitory neurotransmitter [19]. The lives of most people with epilepsy continue to be negatively impacted by gaps in knowledge, diagnosis, and treatment. Concerted actions to address these challenges are urgently needed. Improving the healthcare system and healthcare transformation are the central focus of Saudi Vision 2030. Part of this improvement is to provide proper education to patients regarding their diseases and medication counseling. Patients who are educated about epilepsy are more likely to adhere to their medication regimen, resulting in better disease control, enhanced quality of life, and, ultimately, reduced treatment costs associated with disease progression.

Accordingly, pharmacy students will play a vital future responsibility in educating the patient and improving healthcare services. The Doctor of Pharmacy program (PharmD) at Umm Al Qura University delivers epilepsy content to the pharmacy students prior to the last year in several modules, including physiology (third year), pharmacology (fourth year), and therapeutics modules (fifth year). The students have gained full knowledge about the pharmacology and pathophysiology of epilepsy via a series of lectures and tutorials. The aim of this study was to evaluate the pharmacology and pathophysiology knowledge of senior pharmacy students regarding epilepsy at Umm Al Qura University.

## 2. Materials and Methods

To conduct this study, the authors utilized a cross-sectional research design and created a self-administered questionnaire through Google Forms. The survey was distributed electronically to senior PharmD students at Umm Al-Qura University in Makkah, Saudi Arabia, via email and various social media platforms. Eligible participants in this study include students in the fourth, fifth, and sixth year (intern) in the academic year (2021/2022).

This study excludes students in the first three years of the program and those who are not studying at the College of Pharmacy.

The questionnaire consisted of three sections with a total of 23 questions with multiple choice and true/false options. The first section covers demographic data such as age, gender, and year of the student. The second section deals with knowledge-related questions about the pathophysiology of the disease, including etiology, signs and symptoms, a neurotransmitter responsible for epilepsy, the prevalence of epilepsy, and the neurochemical basis of epilepsy. The third part deals with the pharmacology of epilepsy, including treatment, preferable drugs for status epilepticus, the role of N-methyl-D-aspartate (NMDA) antagonists, drug–drug interactions of epilepsy medication, complementary vitamins used, and current therapeutic approaches.

Prior to data collection, participants were presented with informed consent forms, which emphasized the confidential treatment of their responses and the option to withdraw from the survey at any time. They were also informed of their right to withdraw from this study without incurring any negative consequences. This study was conducted between August and September 2022 and received approval from the biomedical research ethics committee of Umm Al-Qura University (Approval No. HAPO-02-K-012-2022-04-1047).

### Statistical Methodology

Data collected for this study were analyzed using IBM SPSS version 23 (IBM Corp., Armonk, NY, USA) and GraphPad Prism version 9 (GraphPad Software, Inc., San Diego, CA, USA). Descriptive statistics were used to define the socio-demographic characteristics, presented as counts and percentages for categorical variables, and mean and standard deviations for continuous variables. Reliability analysis was performed using Cronbach’s alpha to study the properties of measurement scales and the items that compose them, including the average inter-item correlation. Correlation between variables represented by means was determined using Pearson’s correlation coefficient. Independent *t*-tests and One-way ANOVA tests were used to compare means for two groups and more than two groups, respectively, assuming normal distribution. The null hypothesis was discarded if the *p*-value was less than 0.05.

## 3. Results

In this study, the knowledge of pharmacology and pathophysiology of epilepsy among 211 senior pharmacy students in Saudi Arabia was evaluated. Socio-demographic characteristics showed that participants had an average age of 22.12 ± 1.2 years old (min = 20, max = 26, N = 206), with the majority of them already in their 4th year of the pharmacy course (53.1%, n = 112), followed by around one-third in their 5th year (31.8%, n = 67), and 15.2% (n = 32) in their 6th year. In terms of gender, the participants had an almost equal distribution of males (49.8%, n = 105) and females (50.2%, n = 106).

Results on the knowledge of the participants about the pathophysiology of epilepsy (KPaE) showed that the majority of them answered correctly on items concerning epilepsy’s (a) cause (80.1%, n = 169), (b) definitions (85.3%, n = 180), (c) pathophysiology (91.5%, n = 193), (d) primary symptom (54.0%, n = 114), (e) responsible neurotransmitter (79.1%, n = 167), (f) main cause of neurochemical basis of the abnormal discharges (73.0%, n = 154), (g) generalized seizures examples (51.7%, n = 109), and (h) main description of absence (petit mal seizures)-generalized seizures (51.7%, n = 109), as shown in Table 1. On the other hand, more than half of the students answered incorrectly only on two items, which are (a) example of partial (local and focal) seizures (62.6%, n = 132) and (b) prevalence of epilepsy (80.6%, n = 170). The details on frequency per item of knowledge about the pathophysiology of epilepsy (KPaE) of the studied population can be seen in Supplementary Table S1, and the distribution of correct answers is shown in Supplementary Figure S1.

Table 2 shows the results of the knowledge about the pharmacology of epilepsy (KPhE) of the students, indicating the majority answered correctly on items concerning the (a) main treatment class for epilepsy management (77.7%, n = 164), (b) reason for multiple drug–drug interactions of most antiseizure medications (due to their metabolism through the cytochrome P450 enzyme pathway) (80.1%, n = 169), (c) evaluation of first seizure (usually via complete physical and neurological exam and history) (68.2%, n = 144), and (d) focus of some current therapeutic approaches of antiseizure medications (blocking Na+ channels and Ca++ channels) (71.6%, n = 151). However, more than half of them answered certain items incorrectly more than correctly. These items were related to (a) the preferred use of drugs for status epilepticus (58.8%, n = 124), (b) the inhibitor of excitatory transmission via block excitatory amino acid receptors (78.2%, n = 165), (c) the need for patients especially women taking antiseizure medications to take vitamin B supplements due to thoughts that some antiseizure medications interfere with vitamin B metabolism (82.0%, n = 173), (d) the possible bone density reduction through some antiseizure medications due to induction of hepatic P450 microsomal enzyme (52.6%, n = 111), and (e) the importance of inhibiting GABA activity and activating excitatory glutamate receptors to manage epilepsy (65.9%, n = 139). The details on frequency per item of knowledge about the pharmacology of epilepsy (KPhE) of the studied population can be seen in Supplementary Table S2, and the distribution of correct answers is shown in Supplementary Figure S2.

The mean KPhE and KPaE scores and distribution among students were then assessed (Table 3). Results showed an average score of 6.22 ± 1.9 (min = 0.00, max = 10.00, N = 211) for KPaE and an average score of 4.60 ± 2.1 (min = 0.00, max = 9.00, N = 211) for KPhE. With regard to the distribution of KPaE scores per item, the highest score was found for item 6 (25.1%, n = 53), while the lowest was found in items 0 (1.4%, n = 3) and 10 (1.4%, n = 3). For the distribution of KPhE scores per item, the highest score was reported for item 5 (19.0%, n = 40), while the lowest was for item 9 (0.5%, n = 1).

Reliability analysis for each of the parameters of this study was then performed, showing favorable Cronbach’s alpha values of 0.557 (n = 10) for the KPaE and 0.652 (N = 9) for the KPhE.

The correlation between the knowledge of KPhE and KPaE was then assessed at 0.01 level (2-tailed). Results revealed a significantly positive correlation between KPhE and KPaE (r = 0.518, *p* < 0.001, N = 211).

Correlation between age vs. KPhE and age vs. KPaE were also evaluated, as shown in Table 4. There was a correlation between age and KPaE (r = −0.081, N = 206) and a correlation between age and KPhE (r = 0.095, N = 206). However, the correlations for both compared variables were found to be not significant (*p* > 0.05).

Lastly, the association between KPaE and KPhE scores against factors such as gender and pharmacy year was determined using an independent *t*-test at <0.05 level (Table 5). Results showed that only gender was found to exhibit a significant association against the KPa (*p* < 0.001), suggesting that gender is a significant predictor of KPaE scores. More specifically, females showed significantly higher mean KPaE scores of 6.74 ± 1.8 (N = 106) compared to males, with a mean score of 5.70 ± 1.9 (N = 105). Other comparisons, such as KPaE vs. pharmacy year and KPhE vs. gender and pharmacy year, were found to exhibit no significant association (*p* > 0.05).

## 4. Discussion

Epilepsy is a neurological disease that affects a significant proportion of the global population. It is a complex condition that can manifest in a variety of ways, from brief and sudden seizures to chronic and debilitating convulsions. The management of epilepsy requires a multi-disciplinary approach, with healthcare professionals from various fields working together to provide the best possible care for patients. Pharmacists play a crucial role in this process, as they are responsible for ensuring that patients receive appropriate medication counseling and that their medications are optimized to effectively manage their condition. As such, it is essential that pharmacy students are equipped with a solid foundation of knowledge about the pharmacology and pathophysiology of epilepsy. Therefore, this study sought to assess the level of knowledge among pharmacy students in their fourth, fifth, and sixth years of study to identify any potential gaps in their understanding of epilepsy. The results revealed that 88.2% of students had studied epilepsy at the university.

Epilepsy is experienced by 5 million people annually and reduces the quality of a patient’s life [1]. The respondents in this study did not have background knowledge about how prevalent epilepsy is, as almost half of the total participating students (49%) answered “I do not know”.

The majority of questions in the first section of the questionnaire regarding the knowledge about the pathophysiology of epilepsy (KPaE) were correctly answered, which revealed good knowledge from the pharmacy students, with an overall mean score of 6.22 ± 1.9 out of the maximum score of 10. The findings of this study indicated that most of the respondents had adequate information about the basic aspects of epilepsy pathophysiology, which occurs due to hyperexcitability of neuron-mediated GABA neurotransmitter inhibition and alteration of GABA metabolism that subsequently leads to repeated seizures. Although the main causes of epilepsy are not yet well known, there are several risk factors that may trigger the development of epilepsy, such as genetic factors or environmental factors such as cerebral infections [20]. Around 80% of the respondents referred to genetic predisposition combined with environmental conditions as one of the causes of epilepsy. Interestingly, several studies reported that pharmacy students and well-educated participants believed that epilepsy could be caused by the evil eye [21,22].

The second part of the questionnaire was designed to assess the knowledge about the pharmacology of epilepsy (KPhE). The overall KphE score in this study was 4.6 ± 2.1 out of a maximum score of 9. Based on the questions that were answered correctly, the majority of respondents had basic information related to epilepsy management, including the use of benzodiazepine as a main class for epilepsy management. However, the respondents did not show a comprehensive understanding of how these antiseizure medications work, as they reported that antiseizure medications act by inhibiting GABA, activating glutamate receptors, and irreversibly inhibiting GABA transaminase to block excitatory receptors.

The wide variation of the pharmacokinetic properties of the antiseizure medications is the foundation cause of drug interactions and adverse drug events of these medications [23]. Therefore, part of the pharmacist’s responsibilities is to review the patient’s medication as well as provide special precautions on a case-by-case basis. In the present study, approximately 80% of respondents reported that most antiseizure medications have multiple drug interactions; nevertheless, the majority of them did not demonstrate enough knowledge about the possible adverse events that may occur due to long-term administration of some antiseizure medications that lead to the reduction in bone density as well as the importance of administering vitamin B supplements for women on antiseizure medications, because some antiseizure medications interfere with vitamin B metabolism.

A significant positive correlation between KPaE and KphE was reported in this study, indicating that the student who could answer questions about the pathophysiology of epilepsy correctly and reported an understanding of the basic pathophysiological aspects also answered the questions about the pharmacology of epilepsy correctly. It was also observed that the 4th and 5th year students recorded more KPaE scores compared to the 6th year students; this may be due to the 4th and 5th year students having recently received epilepsy lectures as part of their pharmacy curricula. However, these findings did not show a statistically significant association between KPaE score and pharmacy year among respondents. This finding aligns with a study conducted at a private university in Lebanon, which showed that pharmacy students in their 4th and 5th years had a more comprehensive understanding of epilepsy [24]. It is noteworthy that it was difficult to draw a conclusion out of the comparison between different pharmacy years, as there were low respondent numbers from the 6th year students compared to the other years, as shown in Table 5.

To date, no studies have been conducted in Saudi Arabia to evaluate pharmacy students’ knowledge of the pharmacology and pathophysiology of epilepsy. Therefore, the results of this study will provide baseline information to identify knowledge gaps and assess the sufficiency of education and clinical training provided in the clinical pharmacy program. Ultimately, this will enable the development of a more comprehensive curriculum to ensure that graduates are equipped with the necessary knowledge and skills to effectively manage common neurological conditions such as epilepsy.

### Limitations

This study had a good response among senior pharmacy students; however, the outcomes cannot completely represent the entire body of pharmacy students in Saudi Arabia because the participants were from a single university, and the education of pharmacy programs differs across other universities in Saudi Arabia. Moreover, the present study did not assess the changes in students’ knowledge over time, including before and after commencing their pharmacy practice training.

Another limitation is that this study relied on self-reported data, which may be prone to recall or social desirability bias. Furthermore, the use of an older classification system for epilepsy may limit the clinical relevance of the findings in terms of current treatment approaches. Lastly, this study did not assess the impact of extracurricular activities, prior work experience, or other factors that may influence students’ knowledge of epilepsy, nor did it evaluate practical skills or clinical decision making, which are essential components of pharmacy education and practice.

## 5. Conclusions

In conclusion, this study sheds light on the level of pharmacology and pathophysiology knowledge of epilepsy among senior pharmacy students at Umm Al-Qura University. While the findings indicate an adequate understanding of the basic pathophysiology of epilepsy, the majority of respondents gave a low number of correct responses concerning their knowledge of antiseizure medications. It is crucial to improve healthcare professionals’ knowledge of epilepsy to achieve better patient outcomes, including improved disease management and quality of life for patients with epilepsy.

## Figures and Tables

**Table 1:** Frequency of correct and incorrect responses on the knowledge about pathophysiology of epilepsy (KPaE) among the studied population (N = 211).

Knowledge about Pathophysiology of Epilepsy	Incorrect n (%)	Correct n (%)
From your knowledge what do you think the cause of Epilepsy disease?	42 (19.9)	169 (80.1)
Epilepsy disease can be defined by one of the following statements:	31 (14.7)	180 (85.3)
The pathophysiology of Epilepsy disease is shown to be one of the following	18 (8.5)	193 (91.5)
What is the primary symptom of epilepsy?	97 (46.0)	114 (54.0)
Which neurotransmitter is responsible for epilepsy?	44 (20.9)	167 (79.1)
The neurochemical basis of the abnormal discharges in epilepsy are mainly due to?	57 (27.0)	154 (73.0)
Which of the following is an example of Partial (local, focal) seizures?	132 (62.6)	79 (37.4)
Which of the following is an example of Generalized seizures?	102 (48.3)	109 (51.7)
Which of the following is the main description of Absence (petit-mal seizures)-generalized seizures	102 (48.3)	109 (51.7)
How common is epilepsy?	170 (80.6)	39 (18.5)

**Table 2:** Frequency of correct and incorrect responses on the knowledge about pharmacology of epilepsy (KPhE) among the studied population (N = 211).

Knowledge about Pharmacology of Epilepsy	Incorrect n (%)	Correct n (%)
What is the main treatment class for epilepsy management?	47 (22.3)	164 (77.7)
Which one of the following drugs is preferable use for Status epilepticus?	124 (58.8)	87 (41.2)
Which one following is thought to inhibit the excitatory transmission via block excitatory amino acid receptors?	165 (78.2)	46 (21.8)
Most antiseizure medications have multiple drug–drug interactions because of their metabolism through the cytochrome P450 enzyme pathway?	42 (19.9)	169 (80.1)
Evaluation of first seizure is usually via complete physical and neurological exam and history	67 (31.8)	144 (68.2)
Patients, especially women, taking antiseizure medications should take vitamin B supplements because some antiseizure medications interfere with vitamin B metabolism	173 (82.0)	38 (18.0)
Bone density may be reduced with some antiseizure medications due to induction of hepatic P450 microsomal enzyme	111 (52.6)	100 (47.4)
Some of current therapeutic approaches of antiseizure medications are focusing on blocking Na+ channels and Ca++ channels?	60 (28.4)	151 (71.6)
Therapeutically, Inhibiting GABA activity and activating excitatory glutamate receptors are important to manage epilepsy	139 (65.9)	72 (34.1)

**Table 3:** Mean knowledge about pharmacology of epilepsy (KPhE) and knowledge about pathophysiology of epilepsy (KPaE) scores and distribution among the studied population (N = 211).

Scores	N	Min	Max	Mean	SD
Knowledge of pathophysiology of epilepsy	211	0.00	10.00	6.22	1.9
Knowledge of pharmacology of epilepsy	211	0.00	9.00	4.60	2.1
		Count		%	
Total		211		100.0	
Knowledge of pathophysiology of epilepsy	0	3		1.4	
	2	6		2.8	
	3	10		4.7	
	4	15		7.1	
	5	30		14.2	
	6	53		25.1	
	7	35		16.6	
	8	36		17.1	
	9	20		9.5	
	10	3		1.4	
Knowledge of pharmacology of epilepsy	0	9		4.3	
	1	10		4.7	
	2	13		6.2	
	3	29		13.7	
	4	35		16.6	
	5	40		19.0	
	6	30		14.2	
	7	33		15.6	
	8	11		5.2	
	9	1		0.5	

**Table 4:** Correlation between the age and knowledge about pharmacology of epilepsy (KPhE) and knowledge about pathophysiology of epilepsy (KPaE) among the studied population (N = 211).

Correlations		Age
Knowledge of pathophysiology of epilepsy	r	−0.081
	*p*-value	0.248
	N	206
Knowledge of pharmacology of epilepsy	r	0.095
	*p*-value	0.176
	N	206

Correlation is significant at the 0.01 level (2-tailed).

**Table 5:** Association between the knowledge about pharmacology of epilepsy (KPhE) and knowledge about pathophysiology of epilepsy (KPaE) scores against the gender and pharmacy year among the studied population (N = 211).

Demographics		Total	Knowledge of Pathophysiology of Epilepsy	Knowledge of Pharmacology of Epilepsy
Total		211	6.22 ± 1.9	4.60 ± 2.1
Gender	Male	105	5.70 ± 1.9	4.38 ± 2.1
	Female	106	6.74 ± 1.8	4.82 ± 2.0
*p*-value			<0.001 ^a^	0.122
Pharmacy Year	4th	112	6.31 ± 2.0	6.22 ± 1.9
	5th	67	6.28 ± 1.8	4.30 ± 2.2
	6th	32	5.78 ± 1.8	4.94 ± 1.9
*p*-value			0.372	0.083

^a^ Association is significant using independent *t*-test at <0.05 level.

## Data Availability

Data is unavailable due to privacy.

## Supplementary Materials

The following supporting information can be downloaded at https://www.mdpi.com/article/10.3390/medicina59050848/s1. Figure S1: Distribution of correct answers on the knowledge per item on pathophysiology of epilepsy (KPaE) among the studied population (N = 211); Figure S2: Distribution of correct answers on the knowledge per item on pharmacology of epilepsy (KPhE) among the studied population (N = 211); Table S1: Frequency per item on knowledge about pathophysiology of epilepsy (KPaE) among the studied population (N = 211); Table S2: Frequency per item on knowledge about pharmacology of epilepsy (KPhE) among the studied population (N = 211).
