# Supplementary material for: Evaluation of Pharmacology and Pathophysiology Knowledge of Epilepsy among Senior Pharmacy Students: A Single Center Experience
Source: Medicina (Kaunas). 2023 Apr 28;59(5):848. doi: 10.3390/medicina59050848 (PMC10222504; doi:10.3390/medicina59050848)
Supplement: Supplementary file 1 [file medicina-59-00848-s001.zip › medicina-2281776-supplementary.pdf]

SUPPLEMENTARY TABLES

Supplementary Table S1. Frequency per item on knowledge about pathophysiology of epilepsy (KPaE) among the studied population (N = 211).

**Knowledge about pathophysiology of epilepsy**		**Count**	**%**
Total		211	100.0
Have you studied about Epilepsy disease (AD) in your college?	Yes	186	88.2
	No	20	9.5
	I don't know	5	2.4
From your knowledge what do you think the cause of Epilepsy disease?	Brain stroke	36	17.1
	Genetic predisposition combined with environmental conditions	169	80.1
	I don't know	6	2.8
Epilepsy disease can be defined by one of the following statements	Disorder of the central nervous system	180	85.3
	Clinical manifestation of an abnormal, excessive, hypersynchronous discharge of a population of cortical neurons	22	10.4
	Progressive loss of cognitive function	6	2.8
	I don't know	3	1.4
The pathophysiology of Epilepsy disease is shown to be one of the following	Hyper-excitability of a neuron and/or hyper synchronization	193	91.5
	Hemorrhagic stroke	2	.9
	Ischemic stroke	4	1.9
	I don't know.	12	5.7
What is the primary symptom of epilepsy?	Paralysis	15	7.1
	Convulsions	74	35.1
	Repeated seizures	114	54.0
	I don't know	8	3.8
Which neurotransmitter is responsible for epilepsy?	GABA, or gamma-aminobutyric acid	167	79.1
	Norepinephrine	6	2.8
	Dopamine	11	5.2
	I don't know	27	12.8
The neurochemical basis of the abnormal discharges in epilepsy are mainly due to?	Reduction of epinephrine	4	1.9
	Production of Dopamine	19	9.0
	Alteration of GABA metabolism	154	73.0
	I don't know	34	16.1
Which of the following is an example of Partial (local, focal) seizures?	Tonic-clonic seizure	66	31.3
	Grand-mal epilepsy	20	9.5
	Psychomotor epilepsy	79	37.4
	I don't know	46	21.8
Which of the following is an example of Generalized seizures?	Petitmal epilepsy	109	51.7
	Psychomotor (temporal lobe) epilepsy	35	16.6
	Partial motor epilepsy	17	8.1
	I don't know	50	23.7

Which of the following is the main description of Absence (petit-mal seizures) – generalized seizures	Characterized by changes in consciousness lasting less than 10 seconds	109	51.7
	Characterized by periods of tonic muscle rigidity followed later by jerking of the body (clonus)	31	14.7
	Presented with convulsive or tonic activity corresponding to the neurons involved	13	6.2
	Not sure	58	27.5
How common is epilepsy?	Globally, an estimated 1 million people are diagnosed with epilepsy each year	54	25.8
	Globally, an estimated 5 million people are diagnosed with epilepsy each year	39	18.7
	Globally, an estimated 10 million people are diagnosed with epilepsy each year	13	6.2
	I don't know	103	49.3
	Missing	2	

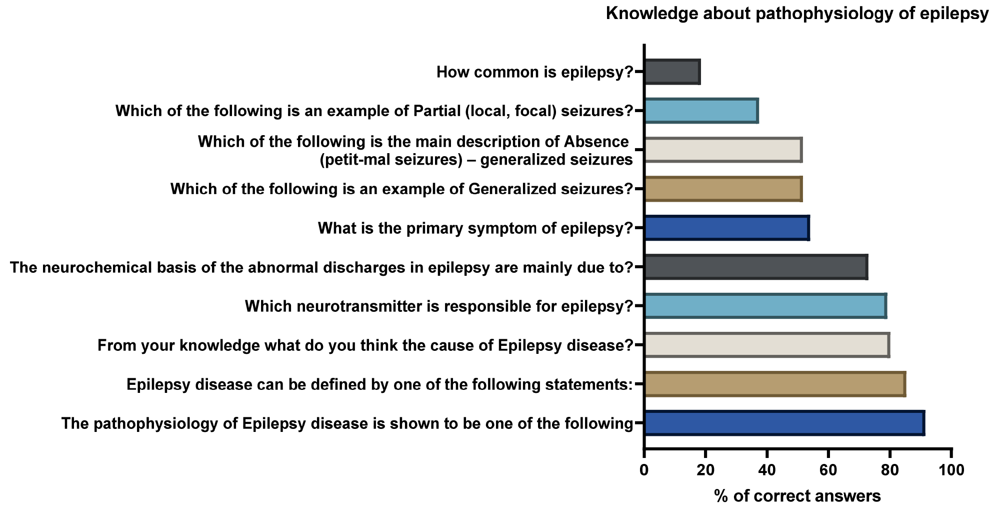

Supplementary Figure S1. Distribution of correct answers on the knowledge per item on pathophysiology of epilepsy (KPaE) among the studied population (N = 211).

Supplementary Table S2. Frequency per item on knowledge about pharmacology of epilepsy (KPhE) among the studied population (N = 211).

**Knowledge about pharmacology of epilepsy**		**Count**	**%**
Total		211	100.0
What is the main treatment class for epilepsy management?	Ergot alkaloids	9	4.3
	Triptans	12	5.7
	Benzodiazepines	164	77.7
	I don't know	26	12.3
Which one of the following drugs is preferable use for Status epilepticus?	Ethosuximide	33	15.6
	Lorazepam	87	41.2
	Lamotrigine	40	19.0
	I don't know	51	24.2
Which one following is thought to inhibit the excitatory transmission via block excitatory amino acid receptors?	N-methyl-D-aspartate (NMDA) antagonists	46	21.8
	Irreversible inhibitors of GABA transaminase (GABAT)	59	28.0
	All of the above	65	30.8
	I don't know	41	19.4
Most antiepileptic medications have multiple drug–drug interactions because of their metabolism through the cytochrome P450 enzyme pathway?	True	169	80.1
	False	11	5.2
	I don't know	31	14.7
Evaluation of the first seizure is usually via a complete physical and neurological exam and history	True	144	68.2
	False	20	9.5
	I don't know	47	22.3
Patients, especially women, taking antiepileptic drugs should take vitamin B supplements because some antiepileptic drugs interfere with vitamin B metabolism	True	86	40.8
	False	38	18.0
	I don't know	87	41.2
Bone density may be reduced with some antiepileptic drugs due to the induction of hepatic P450 microsomal enzyme	True	100	47.4
	False	37	17.5
	I don't know	74	35.1
Some of the current therapeutic approaches of antiepileptic drugs are focusing on blocking Na+ channels and Ca++ channels?	True	151	71.6
	False	15	7.1
	I don't know	45	21.3
Therapeutically, Inhibiting GABA activity and activating excitatory glutamate receptors are important to manage epilepsy	True	106	50.2
	False	72	34.1
	I don't know	33	15.6

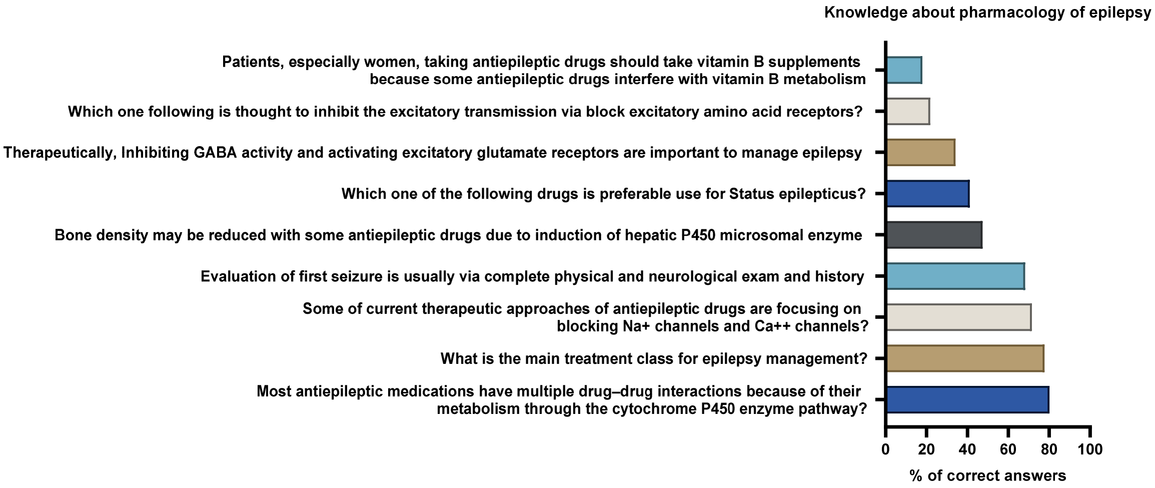

Supplementary Figure S2. Distribution of correct answers on the knowledge per item on pharmacology of epilepsy (KPhE) among the studied population (N = 211).
